# Supplementary material for: Circulating Exosomal Integrin β3 Is Associated with Intracranial Failure and Survival in Lung Cancer Patients Receiving Cranial Irradiation for Brain Metastases: A Prospective Observational Study
Source: Cancers (Basel). 2021 Jan 20;13(3):380. doi: 10.3390/cancers13030380 (PMC7864205; doi:10.3390/cancers13030380)
Supplement: Supplementary file 1 [file cancers-13-00380-s001.pdf]

cancers

MDPI

Supplementary Materials

Circulating Exosomal Integrin β3 Is Associated with Intracranial Failure and Survival in Lung Cancer Patients Receiving Cranial Irradiation for Brain Metastases: A Prospective Observational Study

Guann-Yiing Chen, Jason Chia-Hsien Cheng, Ya-Fang Chen, James Chih-Hsin Yang and Feng-Ming Hsu *

Table S1. Clinical factors associated with intracranial progression-free survival and extracranial progression-free survival.

		Median IC-PFS (month)	95% CI	p-value	Median EC-PFS (month)	95% CI	*p*-Value
Sex	Male	8.0	4.2–9.3	0.11	4.8	3.0–9.1	0.22
	Female	7.9	6.3–19.8		5.7	3.7–10.9	
Number of BM	<5	8.6	5.2–NA	0.93	5.8	2.4–NA	0.86
	5–10	7.2	4.2–NA		3.7	2.8–12.1	
	>10	7.9	5.1–13.7		5.0	3.5–9.3	
LM carcinomatosis	Present	7.4	4.1–9.3	0.93	5.0	3.1–9.1	0.12
	Absent	7.9	5.2–11.9		4.4	2.7–9.5	
Histology	ADC	8.0	7.4–11.7	0.002	5.6	4.8–9.3	0.02
	Non-ADC	3.5	2.4–NA		2.4	1.8–NA	
GPA score	0–1.0	3.6	3.2–NA	0.002	3.3	2.8–NA	0.11
	1.5–2.0	8.0	5.0–13.7		4.8	2.7–9.1	
	2.5–3.0	9.1	7.9–31.3		5.8	5.6–14.3	
	3.5–4.0	19.8	9.3–NA		Not reached	1.4–NA	

Abbreviations: IC-PFS, intracranial progression-free survival; CI, confidence interval; EC-PFS, extracranial progression-free survival; BM, brain metastases; LM, leptomeningeal; ADC, adenocarcinoma; GPA; graded prognostic assessment.

Table S2. Univariate Cox regression analysis of factors associated with overall survival.

	HR	95% CI	*p*-Value
Age (per 1-year increase)	1.03	0.99–1.07	0.12
Sex (female vs. male)	0.62	0.33–1.16	0.14
Histology (adenocarcinoma vs. non-adenocarcinoma)	0.36	0.18–0.73	0.004
Prior SRS (yes vs. no)	0.98	0.81–1.19	0.88
Number of BM (<5, 5–10, >10)	0.99	0.70–1.40	0.94
High GPA (high vs. low)	0.26	0.14–0.48	<0.001
Integrin β3 (per 1 ng/mL increase)	1.15	1.005–1.32	0.04
Integrin β6 (per 1 ng/mL increase)	1.06	0.87–1.29	0.56

Abbreviations: HR, hazard ratio; CI, confidence interval; vs., versus; SRS, stereotactic radiosurgery; BM, brain metastases; GPA; graded prognostic assessment.

Table S3. Univariate subdistribution hazards for factors associated with intracranial failure with death as competing risk.

	sHR	95% CI	*p*-Value
Age (per 1-year increase)	1.01	0.97–1.06	0.54
Sex (female vs. male)	1.1	0.59–2.05	0.76
Histology (adenocarcinoma vs. non-adenocarcinoma)	1.26	0.48–3.32	0.64
Prior SRS (yes vs. no)	1.04	0.99–1.08	0.12
Number of BM (<5, 5–10, >10)	1.05	0.72–1.55	0.8
High GPA (high vs. low)	1.56	0.71–3.42	0.27
Integrin β3 (per 1 ng/mL increase)	1.16	0.995–1.34	0.058
Integrin β6 (per 1 ng/mL increase)	0.995	0.79–1.25	0.96

Abbreviations: sHR, subdistribution hazard ratio; CI, confidence interval; vs., versus; SRS, stereotactic radiosurgery; BM, brain metastases; GPA; graded prognostic assessment.
